# Net primary productivity of forest stands in New Hampshire estimated from Landsat and MODIS satellite data

**DOI:** 10.1186/1750-0680-2-9

**Published:** 2007-10-17

**Authors:** Christopher Potter, Peggy Gross, Vanessa Genovese, Marie-Louise Smith

**Affiliations:** 1 Biospheric Science Branch, NASA Ames Research Center, Moffett Field, CA 94035, USA; 2 Earth System Science and Policy, California State University Monterey Bay, Seaside, CA 93955, USA; 3 US Forest Service, Northern Research Station, Durham, NH 03824, USA

## Abstract

**Background:**

A simulation model that relies on satellite observations of vegetation cover from the Landsat 7 sensor and from the Moderate Resolution Imaging Spectroradiometer (MODIS) was used to estimate net primary productivity (NPP) of forest stands at the Bartlett Experiment Forest (BEF) in the White Mountains of New Hampshire.

**Results:**

Net primary production (NPP) predicted from the NASA-CASA model using 30-meter resolution Landsat inputs showed variations related to both vegetation cover type and elevational effects on mean air temperatures. Overall, the highest predicted NPP from the NASA-CASA model was for deciduous forest cover at low to mid-elevation locations over the landscape. Comparison of the model-predicted annual NPP to the plot-estimated values showed a significant correlation of R^2 ^= 0.5. Stepwise addition of 30-meter resolution elevation data values explained no more than 20% of the residual variation in measured NPP patterns at BEF. Both the Landsat 7 and the 250-meter resolution MODIS derived mean annual NPP predictions for the BEF plot locations were within ± 2.5% of the mean of plot estimates for annual NPP.

**Conclusion:**

Although MODIS imagery cannot capture the spatial details of NPP across the network of closely spaced plot locations as well as Landsat, the MODIS satellite data as inputs to the NASA-CASA model does accurately predict the average annual productivity of a site like the BEF.

## Background

The capacity of forests to sequester carbon from the increasing pool of atmospheric CO_2 _is becoming an issue of central importance for land managers and policy makers. Forested areas that consistently add carbon by growth in ecosystem production are potentially important as current and future sinks for industrial CO_2 _emissions. Conversely, land areas that do not consistently sequester carbon over time may be adding to already rising atmospheric CO_2 _levels from fossil fuel burning sources, Temperate forests recovering from disturbances, such as harvest for wood products or regrowth on abandoned agricultural lands, may represent important sinks globally for CO_2 _[1]. Nonetheless, affordable and rapid methods to understand and quantify the factors controlling the productivity of forest stands over most of the United States still await development.

Although regional- to global-scale relationships between forest carbon uptake and major climatic gradients have been demonstrated in several types of vegetation productivity models [2,3], capturing landscape scale (e.g., less than 1 km^2^), patterns in forest carbon cycles has proven to be a challenge. At a landscape level, the influence of macroclimate is often less important than other sources of spatial variation, including disturbances, hydrology, and soil nutrient supply [4-6]. Nevertheless, several studies have addressed sub-landscape level variation in forest production through coupled applications of remote sensing and ecosystem process models [7-9].

Satellite remote sensing from instruments like Landsat has been applied to forest canopy studies for over a decade [10,11]. The launch of NASA's Terra satellite platform in 1999 with the Moderate Resolution Imaging Spectroradiometer (MODIS) instrument on-board initiated a new era in remote sensing of the Earth system with promising implications for forest carbon research. Direct input of satellite vegetation index "greenness" data from the MODIS sensor into ecosystem simulation models can now be used to estimate spatial variability in monthly net primary production (NPP), biomass accumulation, and litter fall inputs to soil carbon pools [12]. These global MODIS vegetation data sets are available at no charge from NASA data centers, which makes their application for carbon cycle studies most affordable, once proven to be scientifically robust.

### Satellite data

Operational MODIS algorithms were the first to generate the Enhanced Vegetation Index (EVI) [13] as global image coverages from 2000-present. As a successor to the two-channel normalized difference vegetation index (NDVI), EVI represents an optimized vegetation index, whereby the vegetation index isolines in red and near infra-red spectral bands are designed to approximate vegetation biophysical isolines derived from canopy radiative transfer theory and/or measured biophysical and optical relationships. EVI was developed to optimize the greenness signal, or area-averaged canopy photosynthetic capacity, with improved sensitivity in high biomass regions and improved vegetation monitoring through a de-coupling of the canopy background signal and a reduction in atmosphere influences. Houborg and Soegaard [14] found MODIS EVI was able to accurately describe the variation in green biomass, in agriculture areas in Denmark, up to green LAI of 5 (R^2 ^= 0.91). The EVI has been found useful in estimating absorbed PAR related to chlorophyll contents in vegetated canopies [15], and has been shown to be highly correlated with processes that depend on absorbed light, such as gross primary productivity (GPP) [16,17].

In this study, we present the results of the NASA-CASA (Carnegie-Ames-Stanford Approach) model to predict net primary productivity (NPP) fluxes using both Landsat 7 and MODIS imagery as a means to infer variability in temperate forests of the eastern United States. Our NASA-CASA model [18-20] has been designed to estimate monthly patterns in carbon fixation and plant biomass increments using moderate-to-high spatial resolution (30 m to 250 m) satellite remote sensing of surface vegetation characteristics and driven with spatially interpolated climate. The main objectives of this analysis were to (1) evaluate uncertainties in the use of Landsat 30-m imagery collected once during a summer growing season to predict NPP over a forested landscape and (2) to assess whether MODIS image data can capture landscape-level variability in forest production as well as Landsat image data in an eastern U. S. hardwood forest.

### Site description

**Figure 1:**
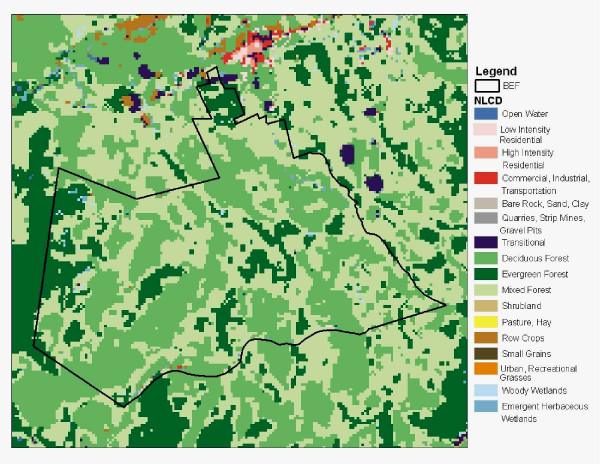
National Land Cover Dataset (NLCD) cover types for the BEF (outline boundary shown).

The Bartlett Experimental Forest – BEF (at approximately Latitude 44.0° N, Longitude 71.3° W) was established in 1932 as a U.S. Forest Service long-term research site. The 25 km^2 ^BEF is located within the White Mountain National Forest, a heavily forested, mountainous region in north-central New Hampshire. The BEF is representative of the larger White Mountain region, having similar vegetative composition (Figure 1), soil types, disturbance histories and topography. As is true of the wider White Mountain region, all but a small portion of the BEF is believed to have a history of logging, although roughly 45% of the land area (particularly at higher elevations) has remained uncut since at least 1890. BEF is an actively managed forest and continues to be subjected to a variety of harvesting practices typical of the region. This is reflected in a range of successional sequences, forest patch sizes, and structural distributions. Natural disturbances affecting portions of the forest include a late 19th century fire, severe wind disturbance resulting from hurricanes in 1938 and 1954, and a damaging ice storm in 1998.

In 1932, a network of permanent forest inventory plots (approximately 0.1 ha each) were established on a regular grid, spaced 200 m by 100 m apart [9]. All trees on most of these plots have been measured by 1-inch diameter classes in at least three time periods, the most recent complete re-measurement at the time of this study being in 2001–2003 [21]. Plot elevations ranged from 220 m to 731 m and represent a range in species composition and successional status. Major tree species encountered were sugar maple (*Acer saccharum*), American beech (*Fagus grandifolia*), white ash (*Fraxinus americana*), paper birch (*Betula papyrifera*), yellow birch (*Betula alleghaniensis*), red maple (*Acer rubrum*), pin cherry (*Prunus pennsylvanica*), eastern hemlock (*Tsuga canadensis*), red spruce (*Picea rubens*), balsam fir (*Abies balsamea*) and eastern white pine (*Pinus strobus*). Most plots contained mixtures of two or more species.

Ollinger and Smith [9] hypothesized that there could be a shift in the importance of moisture versus temperature limitations on forest production from low to high elevations within the White Mountains region. Increases in water availability with elevation may result from several factors in the northeast, including increased precipitation, a decrease in transpiration caused by lower temperatures, and the tendency for mid-elevation soils to be derived from deeper deposits of fine-textured glacial till. Supporting this interpretation, Federer [22] concluded that New Hampshire forests in low-elevation areas probably experience water deficits in most years.

## Results

A comparison of different modeling methods is presented in the following section. Each of these methods use remote sensing data inputs to characterize forest cover attributes that can influence NPP. The principal aim of this analysis is to determine how well satellite image data from Landsat and MODIS can capture landscape-level variability in forest production.

### PnNET-II model for plot-based NPP

Above ground net primary productivity (ANPP, g C m^-2 ^yr^-1^) for BEF plots has been estimated as the sum of wood production plus foliar production, based on plot measurements of tree diameters, litterfall, and allometeric scaling. Using these ANPP estimates for validation, a complete forest NPP (above- and below-ground) data set was generated from the forest process model PnET-II [23], initialized with high spectral resolution imagery from AVIRIS (Airborne Visible/Infrared Imaging Spectrometer) for foliar N estimation [9]. PnET-II requires a number of other input parameters summarizing vegetation and site characteristics, along with monthly climatic data. Vegetation parameters include foliar N, leaf retention time and growing-degree day variables describing the phenology of leaf production and senescence. Required climatic and soil inputs include temperature, precipitation, photosynthetically-active radiation (PAR), and soil water holding capacity (WHC). For pixel-by-pixel application at BEF, PnET-II was run in conjunction with image-derived foliar N estimates and a 20-m resolution digital elevation model (DEM). For each 20-m pixel estimate of NPP, geographic coordinates and elevation were extracted and used to estimate maximum and minimum temperature, vapor pressure, precipitation, and PAR.

### CASA modeling methods for NPP

As documented in by Potter [19], the monthly NPP flux, defined as net fixation of CO_2 _by vegetation, is computed in NASA-CASA on the basis of light-use efficiency [24]. Monthly production of plant biomass is estimated as a product of time-varying surface solar irradiance, Sr, and EVI from the MODIS or Landsat satellite data, plus a constant light utilization efficiency term (emax) that is modified by time-varying stress scalar terms for temperature (T) and moisture (W) effects (Equation 1).

NPP = S_r _EVI e_max _T W

**Figure 2:**
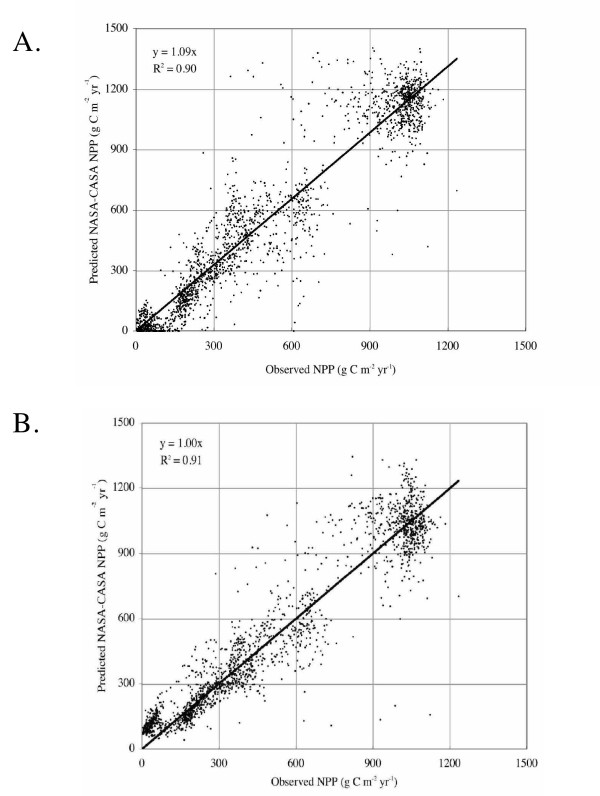
Comparison of annual observed NPP to predicted values from the NASA-CASA model (driven by 0.5° data inputs). (a) Inputs from AVHRR-FPAR 1982 and climate means from New et al. (2000), (b) Inputs from MODIS-EVI 2001 and climate from NCEP reanalysis products for 2001. Both figures include the 1:1 regression line. The data set of more than 1900 observed NPP points was compiled for the Ecosystem Model-Data Intercomparison (EMDI) activity by the Global Primary Productivity Data Initiative (GPPDI) working groups of the International Geosphere Biosphere Program Data and Information System [34,35].

The default emax term is set uniformly at 0.39 g C MJ^-1 ^PAR, a value that derives from calibration of predicted annual NPP to previous field estimates [2]. This model calibration has been validated globally by comparing predicted annual NPP to more than 1900 field measurements of NPP (Figure 2a). Interannual NPP fluxes from the CASA model have been reported [25] and validated against multi-year estimates of NPP from field stations and tree rings [26]. Our NASA-CASA model has been validated against field-based measurements of NPP fluxes and carbon pool sizes at multiple boreal forest sites in North America [18,26-28] and against atmospheric inverse model estimates of global carbon fluxes [20].

The T stress scalar is computed with reference to derivation of an optimal seasonal temperature (Topt) for plant production. Over large areas, the Topt setting will vary by latitude and longitude, ranging from near 0°C in the Arctic to the middle thirties in low latitude deserts. The W stress scalar is estimated from monthly water deficits, based on a comparison of moisture supply (precipitation) to potential evapotranspiration (PET) demand using the method of Thornthwaite and Mather [29]. The Moderate Resolution Imaging Spectroradiometer (MODIS) 1-km land cover map [30] was used to specify the predominant land cover class for the W term in each pixel as either forest or non-forest classes.

Whereas previous versions of the NASA-CASA model [2,18] used NDVI bands to approximate FPAR, the current model version instead has been calibrated to use MODIS EVI datasets as direct inputs to Equation 1 above. In long-term (1982 to 2004) simulations, continuity between AVHRR and MODIS sensor data for inputs to NASA-CASA is an issue that must be addressed by recalibration of annual NPP results post 2000. NASA-CASA model predictions with 2001 monthly MODIS EVI inputs have been adjusted using the same set of field measurements of NPP shown in Figure 2a, to which the model was previously calibrated for a best linear fit to AVHRR inputs [20]. To best match predictions with previously measured NPP estimates at the global scale (Figure 2b), the model emax term for 2001 MODIS EVI inputs should be reset to 0.55 g C MJ^-1 ^PAR, a value that is globally 42% higher than previously used in the model for AVHRR-driven NPP predictions from 1982–1998 [20]. The regression coefficient (with line intercept forced through zero) of R^2 ^= 0.91 for this NPP recalibration to 2001 MODIS EVI inputs was statistically significant (*p *< 0.01).

In the following section, an evaluation procedure at the landscape scale is presented for the NASA-CASA model at BEF using growing season EVI computed from Landsat 7 Enhanced Thematic Mapper (ETM+) imagery at a 30-m gridded spatial resolution. We hypothesize that to match estimated NPP across BEF sample plots, the spatial detail provided by Landsat imagery collected during the summer period when leaf area is near maximum for the year will compensate for the lack of repeated EVI sampling during each month of the year (that, for example, is provided by the MODIS sensor, even though MODIS data is at a coarser 250-m minimum resolution). The rationale behind this hypothesis is that monthly climate inputs to the NASA-CASA model that uses a single Landsat image may be sufficient to capture the canopy phenology patterns throughout any given year that MODIS monthly satellite observations are used to capture. Furthermore, 30-m elevation and Landsat data inputs to NASA-CASA may serve as proxies for local weather variation effects on NPP.

**Figure 3:**
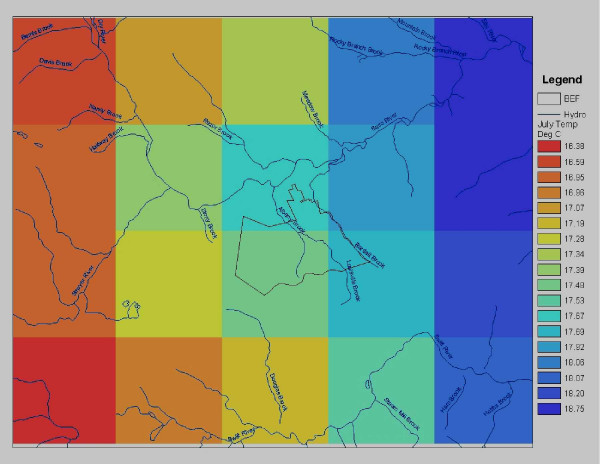
PRISM [31] mean monthly temperature grid (20 km × 16 km) for the BEF area (outlined at center) showing the gradient of relatively cooler to warmer conditions moving from higher elevation to lower elevation, predominantly west to east (left to right).

In all the NASA-CASA simulation results presented for the BEF in this study, 4-km resolution spatial grids from PRISM (Parameter-elevation Regressions on Independent Slopes Model) [31] were used for precipitation, average maximum temperature, and average minimum temperature. These 4-km climatologies were derived from U. S. weather stations records interpolated first into 30 arc-second data sets. PRISM is unique in that it incorporates a spatial climate knowledge base that accounts for topographic influences such as rain shadows, temperature inversions, and coastal effects, in the climate mapping process. An example of the PRISM monthly temperature product for the BEF area is shown in Figure 3, wherein higher elevation areas in the western portion of the area typically have cooler summertime temperatures than the lower elevation portions of the area.

### CASA model NPP using Landsat EVI inputs

**Figure 4:**
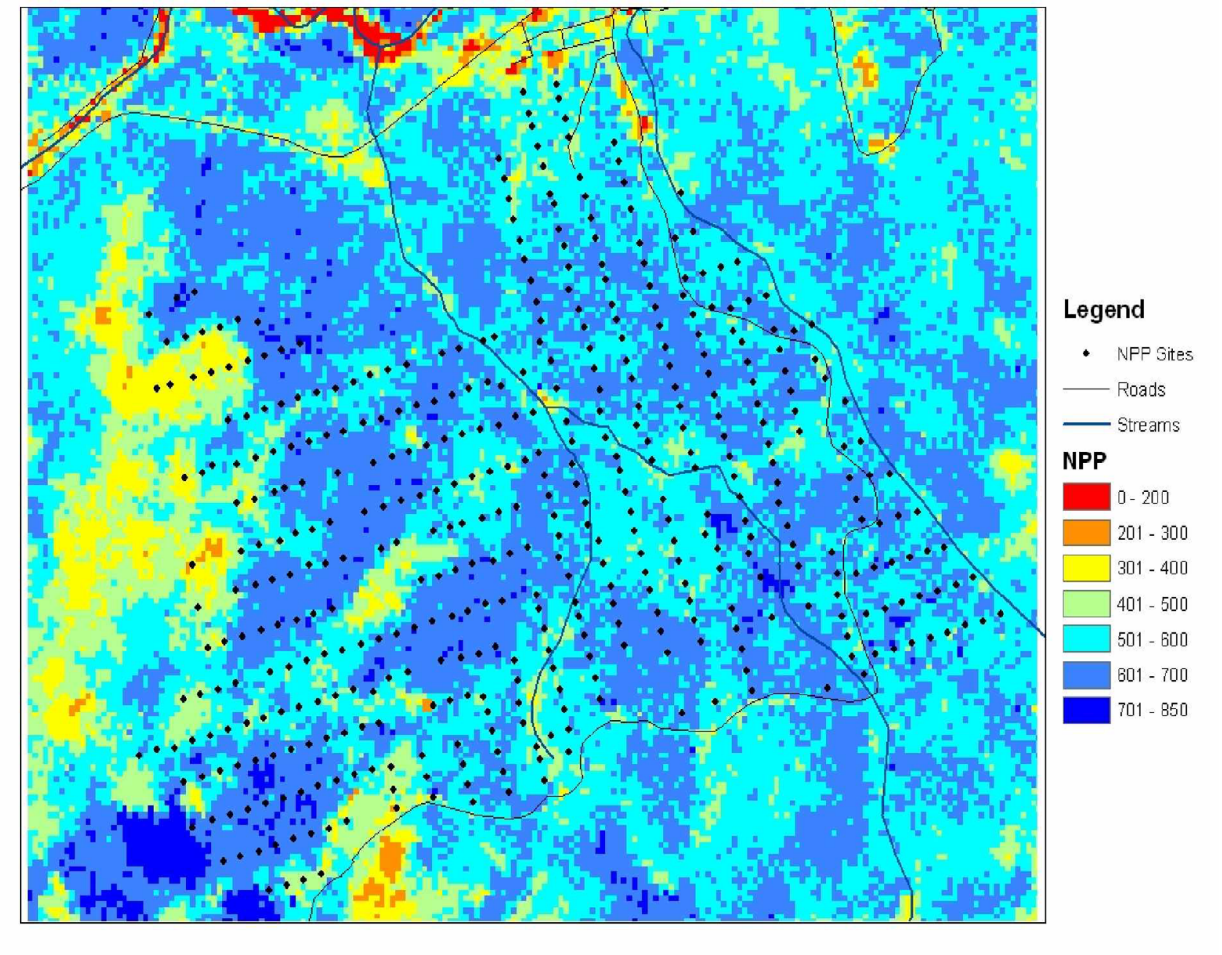
CASA model NPP for the BEF area using Landsat 7 EVI and monthly PRISM climate inputs from 2001. Plot measurement sites are shown as a point grid.

The Landsat 7 imagery used for this NASA-CASA modeling was collected on August 29, 2001. Predicted NPP from the NASA-CASA model for the BEF (Figure 4) shows variations related to both vegetation cover type (Figure 1) and elevational effects on mean air temperatures (Figure 3). Overall, the highest predicted NPP from the NASA-CASA model was for deciduous forest cover at low to mid-elevation locations over the landscape.

**Figure 5:**
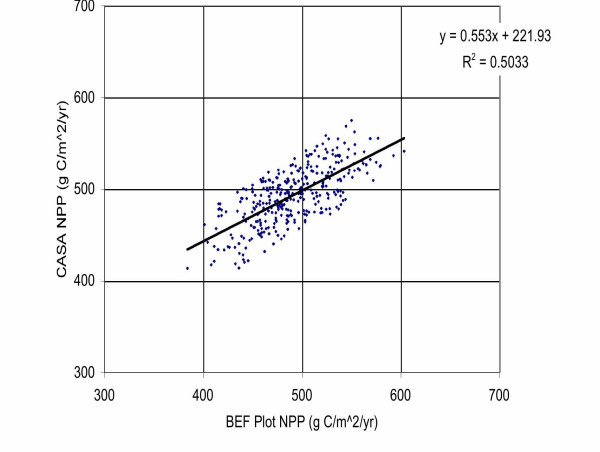
Plot measurement NPP for the BEF area versus CASA model NPP using Landsat 7 EVI and monthly PRISM climate inputs from 2001.

The Landsat 30-m resolution grid coverage was used to stratify 3 × 3 pixel areas around each BEF plot location into comparable predictions of annual NPP from the CASA model. Comparison of the NASA-CASA model-predicted annual NPP to the BEF-estimated values (Figure 5) showed a significant correlation of R^2 ^= 0.5 (*p *< 0.05) following the removal of 90 outlier plot values that were determined to have suffered heavy damage to forest stands during winter ice storms of January, 1998. The AVIRIS imagery used as an input to BEP plot NPP estimation was collected in 1997 and much of the Forest Service field data reflects pre-1997 tree status as well. Because we have used a more recent (i.e., 2001) Landsat 7 image for this study, NASA-CASA predicted NPP should be a more updated representation of BEF plot productivity.

In an attempt to improve upon these initial results, we subdivided the BEF plots into three different forest types: Deciduous, Evergreen, and Mixed, defined by the National Land Cover Dataset (NLCD) [32], 1999; (Figure 1) at 30-m resolution. Separate correlation results for NASA-CASA predictions versus the measured plot NPP were generated for each NLCD forest type. Previously published studies for the BEF [9,21] suggest that Deciduous stands have higher nitrogen in their tree leaves and hence higher average annual NPP even at the same leaf cover level as the Evergreen stands. We find that the simple breakout by NLCD forest type did not improve correlation statistic values, but did result in reduced variations around the mean and hence the newly stratified correlation line falls closer to a 1:1 line for the Evergreen forest stands (data not shown). This closer 1:1 match was not observed for the Deciduous stands, which presumably grow on a wider range of soil nutrient conditions.

Previously published studies also suggested that low elevation stands at BEF tend to have lower NPP than mid- to higher elevation stands [9], a trend that runs counter to most other forested locations with rugged terrain. Forest soils at low elevation at the BEF can have lower moisture availability compared to mid-elevation soils. We hypothesized therefore that including elevation as an independent variable in the regression analysis would explain a significant additional portion of the unexplained variance of estimated NPP in Figure 5. This hypothesis was tested using stepwise multiple linear regression, from which it was determined that addition of 30-meter elevation data values increased the R^2 ^coefficient by 0.17. The addition of 30-meter estimates for aspect direction (0–360°) in the stepwise regression increased the R^2 ^coefficient by another 0.02, leading to a final R^2 ^= 0.69 for correlation of NASA-CASA predicted NPP plus topographic variables with the measured NPP patterns at BEF plots.

**Figure 6:**
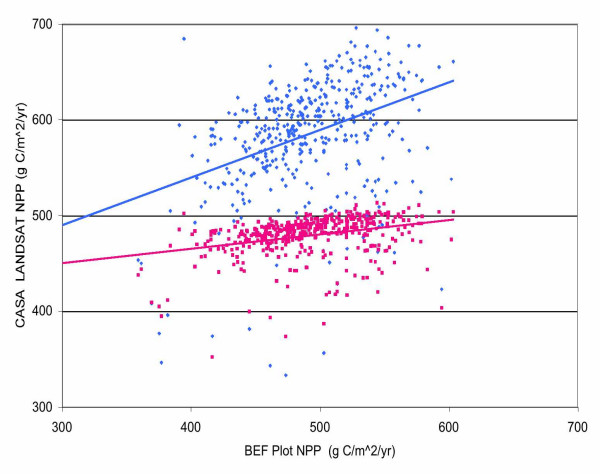
Plot measurement NPP for the BEF area versus CASA model NPP using Landsat 7 EVI (blue diamond symbols) or NDVI (red square symbols) and monthly PRISM climate inputs from 2001. Best-fit regression lines are shown for both EVI and NDVI results.

Because we can compute both NDVI and EVI from the 2001 Landsat 7 bands, it is possible to compare NASA-CASA NPP results to the BEF NPP estimates using either vegetation index from the satellite imagery. It is evident that NDVI is saturating at the high end of the greenness (i.e., actual leaf area index – LAI) range, whereas EVI is saturating to a lower degree in this manner and instead spreads out the greenness inputs to NASA-CASA more linearly over the entire range of NPP estimates at the BEF (Figure 6). The result is a closer fit to the predicted 1:1 line using the EVI compared to the NDVI as the NASA-CASA model input variable (with the same climate and topographic settings) over the BEF coverage area.

### CASA Model NPP Comparison using MODIS EVI

**Figure 7:**
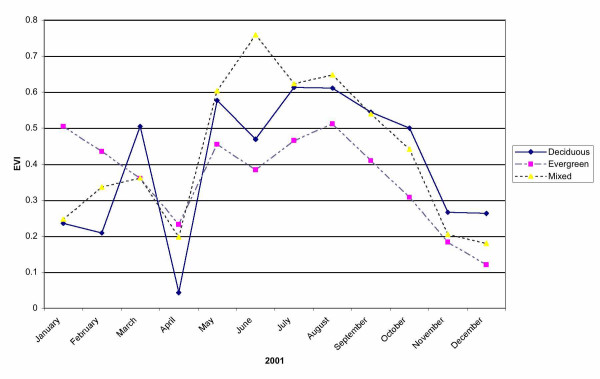
MODIS time series profiles for EVI at three 250-m pixel locations selected as representative of nearly uniform coverage (> 90%) of Deciduous, Coniferous, and Mixed forest classes at the BEF.

MODIS EVI composite images for all months of 2001 were used in place of the single Landsat 7 EVI imagery to predict annual NPP at the BEF. These continuous gridded observations of canopy greenness cover from the MODIS instrument have been collected to improve the tracking of canopy green-up and green-down over a growing season, particularly for Deciduous and Mixed forest stand types. MODIS time series profiles for EVI at three 250-m pixel locations within the BEF could be identified at locations of nearly uniform coverage (> 90%) of single forest classes from the NLCD map. These profiles showed that Deciduous and Mixed forest types reached maximum greenness cover levels by end of July, whereas Coniferous forest types increased in greenness cover levels until mid-August (Figure 7). Compared to the other two forest types, greenness cover in Coniferous forest types consistently remained about 20% lower from May through December and then increased to about 10% higher during January and February.

Nevertheless, using fully gridded 250-m MODIS EVI inputs to the NASA-CASA model (with no sub-sampling bias), comparison of predicted annual NPP to the BEF-estimated plot NPP values showed no significant correlation result (at *p *< 0.1), regardless of whether the entire collection of plot estimates were considered together, or were separated into predominantly Deciduous, Coniferous, and Mixed forest classes (data not shown). This implies that the relatively coarse spatial resolution of the MODIS 250-m EVI imagery cannot capture landscape-level variability in tree production in the same way that 30-m Landsat image data can for this eastern hardwood forest location.

On the other hand, both the MODIS and Landsat 7 derived mean annual NPP predictions for the BEF plot locations were within ± 2.5% of the mean of PnET-based plot estimates for annual NPP. The mean annual NPP prediction from the NASA-CASA model using MODIS 250-m EVI imagery for the BEF was 503 g C m^-2 ^yr^-1^, and therefore was slightly closer to the mean of all PnET-based plot measurements for annual NPP of 492 g C m^-2 ^yr^-1 ^than was the mean annual NPP prediction from the NASA-CASA model using Landsat 30-m EVI imagery of 479 g C m^-2 ^yr^-1^. This finding suggests that, although MODIS 250-m EVI imagery cannot capture the spatial details of NPP across the network of closely spaced plot locations, it does accurately predict the average annual productivity of a site like the BEF.

## Conclusion

Evidence from this modeling study indicates that the satellite-observed canopy greenness represented by the EVI is useful as a variable to help account for carbon sinks in northeastern forest ecosystems. When controlling for elevation, the NASA-CASA model using inputs of 30-m resolution Landsat 7 EVI for the month of peak growing season temperatures captures a substantial portion (> 67%) of landscape scale variation in the controls (such as foliar N content and moisture stress effects) on annual NPP at BEF. Nonetheless, a major limitation of Landsat imagery as a routine input to forest productivity models is the relatively low number of cloud-free scenes that can be collected over the course of a growing season in most temperate and humid climate zones. With a return schedule of approximately 16 days, the probability of collecting one cloud-free Landsat image per month of the year is low.

In comparison, MODIS imagery can be used in the NASA-CASA model to generate accurate predictions of the average forest NPP for a site the size of the BEF. Because MODIS provides global daily coverage of the entire Earth surface, these 250-meter resolution images can be composited over several weeks to provide an economical and rapid method to capture the regional-scale factors controlling seasonal productivity over most forested areas of the United States. NASA-CASA model predictions from MODIS 250-meter resolution data inputs are best suited to assessments of large forested tracts of land where stand ages are relatively uniform.

A next logical step in this methodology of using satellite image data as inputs to models of forest production at the landscape scale is to combine Landsat and MODIS data sets and enhance the advantages of both sensor measurements. We are investigating techniques to interpolate between dates of 30-meter resolution Landsat EVI data values by an appropriate sampling of monthly MODIS EVI values at selected locations that can represent much of the spatial variability in forest stand types and canopy properties detected in the higher resolution Landsat imagery.

## Competing interests

The author(s) declare that they have no competing interests.

## Authors' contributions

All authors have made substantial contributions to the analysis and interpretation of data, have been involved in drafting the manuscript and revising it critically for important intellectual content, and have given final approval of the version to be published.
